# Lamivudine/Adefovir Treatment Increases the Rate of Spontaneous Mutation of Hepatitis B Virus in Patients

**DOI:** 10.1371/journal.pone.0163363

**Published:** 2016-09-20

**Authors:** Marianoel Pereira-Gómez, Juan-Vicente Bou, Iván Andreu, Rafael Sanjuán

**Affiliations:** 1 Institute for Integrative Systems Biology (I2SysBio), Universitat de València, València, Spain; 2 Departament de Genètica, Universitat de València, València, Spain; Defence Research Laboratory, INDIA

## Abstract

The high levels of genetic diversity shown by hepatitis B virus (HBV) are commonly attributed to the low fidelity of its polymerase. However, the rate of spontaneous mutation of human HBV in vivo is currently unknown. Here, based on the evolutionary principle that the population frequency of lethal mutations equals the rate at which they are produced, we have estimated the mutation rate of HBV in vivo by scoring premature stop codons in 621 publicly available, full-length, molecular clone sequences derived from patients. This yielded an estimate of 8.7 × 10^−5^ spontaneous mutations per nucleotide per cell infection in untreated patients, which should be taken as an upper limit estimate because PCR errors and/or lack of effective lethality may inflate observed mutation frequencies. We found that, in patients undergoing lamivudine/adefovir treatment, the HBV mutation rate was elevated by more than sixfold, revealing a mutagenic effect of this treatment. Genome-wide analysis of single-nucleotide polymorphisms indicated that lamivudine/adefovir treatment increases the fraction of A/T-to-G/C base substitutions, consistent with recent work showing similar effects of lamivudine in cellular DNA. Based on these data, the rate at which HBV produces new genetic variants in treated patients is similar to or even higher than in RNA viruses.

## Introduction

With around 250 million people infected worldwide, hepatitis B virus (HBV) constitutes a major cause of cirrhosis, liver failure and hepatocellular carcinoma [1]. HBV has a small (3.2 kb), partially double-stranded, circular DNA virus that is primarily encapsidated as a pre-genomic RNA that undergoes reverse transcription after encapsidation [2,3]. The HBV genome is extremely compact, with four, partially or fully overlapped open reading frames (C, S, P, and X). The preC and C reading frames encode the *e* antigen (HBeAg) and core protein (HBcAg) respectively, the preS and S reading frames encode three forms of surface proteins (HBsAg) sharing the C-terminus (small, middle and large), the P gene encodes the viral polymerase, which acts both as reverse transcriptase (RT) and DNA-dependent DNA polymerase, and the X gene encodes a transcriptional trans-activator protein. HBV treatment consists of immune modulators (interferon) combined with nucleoside analogues whose primary effect is to inhibit the viral polymerase [4,5]. However, treatment typically fails to clear the virus, which remains active in the form covalently closed circular DNA within hepatocyte nuclei [6], and long-term treatment with nucleoside analogues often selects for drug-resistant mutations in the HBV polymerase [7].

HBV shows a molecular evolutionary rate on the order of 10^−4^ nucleotide substitutions per site per year (s/s/y) at the epidemiological scale [8,9], and of 10^−5^–10^−4^ s/s/y at the intra-patient level [10,11]. This rate is notably higher than those of most non-reverse transcribing DNA viruses, and similar to those of RNA viruses and retroviruses [12]. It is commonly accepted that such fast evolution is determined ultimately by the low replication fidelity the HBV polymerase which, similar to other RTs, lacks 3´exonclease proofreading activity [13] and produces frequent replication errors, resulting in highly diverse viral populations [14–17]. Despite growing interest in studying the relationship between HBV genetic variation and viral pathogenesis, as well as its implications for viral detection, prevention, treatment and prognosis, the rate of spontaneous mutation of human HBV has not been determined. Notice that, whereas the molecular evolutionary rate refers to a population genetics process, the rate of spontaneous mutation refers to a biochemical process and, therefore, these two rates should not be confused. The former describes the fixation of new alleles in a population and is determined by factors such as selection and random drift, whereas the latter is defined as the probability that new mutations appear per round of genome copying, or per cell infection cycle, and is determined by factors such as polymerase fidelity, DNA/RNA editing, and spontaneous damage. Early work with the related duck HBV estimated the reversion frequency of a single deleterious G-to-A nucleotide substitution [18] and, after correcting for several possible confounders such selection and the unknown number of rounds of replication, this yielded a mutation rate estimate of 2 × 10^−5^ per nucleotide per cell infection cycle (m/n/c) [19]. However, the reliability of this value was compromised by the fact that only a single genome site was analyzed.

Another unaddressed question is the effect of treatment on the HBV mutation rate. Broad-spectrum nucleoside analogues such as ribavirin are known to mutagenize RNA viruses [20–25]. Similarly, nucleoside analogue RT inhibitors such as lamivudine and AZT have a direct mutagenic effect on HIV-1 in cell culture, in addition to their inhibitory effects [26]. Moreover, treatment with nucleoside analogues can select for resistance mutations that modify the replication fidelity of the HIV-1 RT, producing an additional, indirect effect on the viral mutation rate [27,28]. In HBV, analysis of nucleotide misincorporation kinetics in vitro showed that typical lamivudine-resistant polymerase variants such as M204I and M204V display increased replication fidelity in the absence of the drug [13], suggesting a mutagenic effect for lamivudine in patients. Nucleoside analogue RT inhibitors including lamivudine have also been found to be mutagenic in different cell lines, as shown by analysis of reporter cellular genes [29,30].

Rates of spontaneous mutation can be inferred in vivo from intra-host sequence diversity data using the lethal mutation method, which is based on the principle that the population frequency of mutations that abolish viral infectivity (lethal mutations) equals the rate at which they are produced, as these cannot be inherited [31]. Using premature stop codons in essential genes as a proxy to lethal mutations, we and others have previously used this method for estimating the mutation rate of hepatitis C virus (HCV) [21,32] and HIV-1 [33] from patient-derived sequences. Here, we applied the lethal mutation method to publicly available, full-length, molecular clone HBV sequences derived from untreated patients, as well as from patients undergoing long-term lamivudine/adefovir combination therapy. We obtained an estimated spontaneous rate on the order of 10^−5^ m/n/c for HBV, albeit with ample variations among HBV reading frames. Furthermore, we show that lamivudine/adefovir elevates the spontaneous HBV mutation rate by at least sixfold. Analysis of intra-host sequence diversity indicated that lamivudine/adefovir treatment produces a shift in the HBV mutation spectrum whereby A-to-G and the complementary T-to-C base transitions are specifically elevated.

## Material and Methods

### Data

We searched molecular clone full-length sequences deposited in GenBank. Publications were first browsed in PubMed based on the following electronic search strategy: ((hepatitis b virus) AND genome*) AND (((clone*) OR cloning) OR full-length). This allowed us to retrieve a large number of articles, which were then inspected to collect only sequences that explicitly included the method used to obtain HBV molecular clones. Also, if fewer than three sequences per patient were analyzed, these were discarded. All sequences corresponded to plasma or serum samples, except for AF182805 and AF182804, which were obtained from a resected peritumor liver tissue and a hepatocellular carcinoma, respectively. Sequences were aligned using the MUSCLE algorithm (www.drive5.com/muscle) and refined further by visual inspection. A neighbor-joining tree was obtained with MEGA6 (www.megasoftware.net) to assess whether patients corresponded to monophyletic sequence groups, and the HBV genotype was determined using the genotyping tool available from the French HBV database (hbvdb.ibcp.fr). Sequences were visualized with MEGA6 and Jalview (www.jalview.org) and phylogenetic trees were edited with TreeDyn (www.treedyn.org).

### Mutation rate estimation by the lethal mutation method

The number of sites that can mutate to a stop codon after a single base substitution (non-sense mutation targets, NSMTs), as well as the observed premature stop codons, were extracted from sequences. For each stop codon mutation, we identified the NSMT and calculated a correction factor *C* that accounts for the fact that there are three possible substitutions for each base (*C* = 3) and that, in some NSMTs, two of these substitutions lead to a stop codon (*C* = 3/2) [21]. These steps were performed using a custom R script. The preS1/preS2 and preC regions were excluded from the analysis because stop codons at these regions are not necessarily lethal and have been implicated in immune escape and pathogenesis [34,35]. For the other reading frames, we discarded sequences in which the stop codon was located in the 5% end of the protein, that lacked AUG initiation codon, with frameshift mutations, or with more than one stop codon. The mutation rate was calculated as the sum of stop codons, each multiplied by the correction factor *C*, divided by the total number of NSMTs.

### Overall mutation spectrum

The consensus sequence of each patient was obtained and differences relative to the consensus were counted for each sequence within this patient and for each possible substitution type, using a custom R script.

## Results

**Fig 1:**
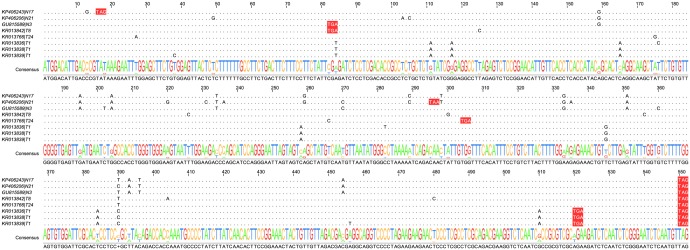
Premature stop-codon mutations found in HBV gene C. Sequence labels indicate the GenBank accession and the patient code according to Table 1. Below is shown the consensus sequence and the colored sequence logo summarizes variability at each position. Only variable sites are shown in the patient sequences, and stop codons are highlighted in red. As also indicated on Table 2, some stop codons appeared repeatedly in the same patient, or even in different patients. Similar alignments could be obtained for the other HBV genes by retrieving GenBank accession numbers from S1 Table.

We analyzed 621 full-length molecular clone sequences obtained from seven studies [36–42], of which 451 corresponded to 22 untreated patients and 170 to 24 patients receiving long-term lamivudine/adefovir therapy (Table 1). We initially scored premature stop codons in the S, C, P, and X genes as a proxy for lethal mutations. In total, we found 23 premature stops in the S, C, P, and X genes in sequences derived from untreated patients (S1 Table). Fig 1 provides an alignment of premature stop codon-containing sequences for gene C. Mutations were not evenly distributed along the genome, since the S gene accumulated 18 of the 23 mutations (Table 2). Based on these anomalously high counts and on previous findings suggesting that S proteins with stop codons can be maintained at high frequencies in patients [43–46], we removed this gene from subsequent analyses. Considering the counts for genes C, P, and X, the estimated HBV mutation rate was 8.7 × 10^−5^ per nucleotide per cell infection cycle. This value is within the same order of magnitude as the rate reported for duck HBV and is also within the range of mutation rates reported for RNA viruses and retroviruses [18,19].

**Table 1:** Dataset used and number of sequences analyzed in each patient.

Dataset	Treated	HBV genotype	Patient IDs (sequences)^a^	Ref.
**I**^b^	No	B	N1 (31), N2 (31), N3 (33), N4 (30), N5 (30), N6 (35), N7 (35)	[36]
**II**^c^	No	A/C/G	N8 (9), N9 (5)	[37]
**III**^d^	No	C	N10 (20)	[38]
**IV**^e^	No	C	N11 (4)	[39]
**V**^f^	No	C	N12 (3), N13 (4), N14 (4), N15 (3)	[41]
**VI**^g^	No	B	N16 (24), N17 (14), N18(44), N19 (11), N20 (26), N21 (37), N22 (18)	[40]
**VII**^h^	Yes	C	T1 (9), T2 (9), T3 (8), T4 (5), T5 (3), T6 (9), T7 (6), T8 (8), T9 (9), T10 (4), T11 (9), T12 (6), T13 (7), T14 (10), T15 (8), T16 (9), T17 (6), T18 (10), T19 (5), T20 (3), T21(7), T22 (3), T23 (8), T24(9)	[42]

^a^Patient IDs created for this study, number of sequences retrieved for each patient shown in parentheses.

^b^Chronically infected subjects infected for >10 years; Stratagene Easy-A High Fidelity PCR DNApol was used (error rate: 1.3 × 10^−6^). Accessions: GU815646-54, GU815656-70, GU815672-8, GU815548-78, GU815579, GU815581, GU815583-GU815591, GU815593-614, GU815616-45, GU815679-81, GU815684-9, GU815691-710, GU815711-3, GU815715-46, GU815747-57, GU815759-62, GU815764-83.

^c^Chronically infected man (33 years old) and woman (24 years old); Taq pol was used for PCR (error rate: 8 ×10^−6^). Recombinant viral genotype. Accessions: HQ231877-85, KF425553-57.

^d^Chronically infected 31 years-old woman infected for >7 years; ex Tal pol was used for PCR (error rate: 1.8 × 10^−6^). Accessions: DQ377160-5, EU306713, EU306722, EU306724-9, EU439005, EU439008, EU439010-2, EU439025.

^e^Chronically infected 48 years-old man, with two sampling dates; Taq pol was used for PCR (error rate: 8 × 10^−6^). Accessions: AF182802-5.

^f^Three of four patients had occult hepatitis; nested PCR was required; LA Taq pol was used for PCR (error rate: 1.3 × 10^−6^). Accessions: EU916223-5, EU916226-9, EU916215-8, EU916239-41.

^g^Chronically infected subjects through vertical transmission; Stratagene Easy-A High Fidelity PCR DNApol was used (error rate: 1.3 × 10^−6^). Accessions: KP406220-43, KP406162-75, KP406176-219, KP406244-54, KP406255-80, KP406281-317, KP406318-335.

^h^Chronically infected subjects treated for over two years; LA Taq was used for PCR (error rate: 1.3 × 10^−6^). Accessions: KR013835-41, KR014021, KR014023, KR013820-6, KR013995, KR013997, KR013854-7, KR014035-9, KR013770, KR013900, KR013902-3, KR013905, KR013817-19, KR013878-9, KR013881-6, KR013890, KR013777-8, KR013913, KR013915, KR013919, KR013923, KR013842-9, KR013792-6, KR013954, KR013956-8, KR013850-3, KR013799-801, KR013968-9, KR013972, KR013974-5, KR013977, KR013858, KR014046, KR014048-9, KR013771-4, KR013776, KR013908-9, KR013809-16, KR013985-6, KR013828-34, KR014019, KR013859-62, KR014055-8, KR014060, KR013872-7, KR013802-8, KR013982-4, KR013827, KR013998, KR014000, KR014006-7, KR013791, KR013943, KR013948, KR013788-90, KR013938-9, KR013941-2, KR013797-8, KR013961, KR013779-86, KR013761-9.

**Table 2:** Summary of stop codons found in each HBV gene.

Gene	Treated	NSMTs	Stops (total)	Rate (total)	Stops (unique)	Rate(unique)
**S**	No	36197	18	1.5 × 10^−3^	9	7.5 × 10^−4^
**P**		110138	1	2.7 × 10^−5^	1	2.7 × 10^−5^
**X**		17593	1	1.7 × 10^−4^	1	1.7 × 10^−4^
**C**		27062	3	2.8 × 10^−4^	3	2.8 × 10^−4^
**S**	Yes	12928	17	3.8 × 10^−3^	5	1.0 × 10^−3^
**P**		40179	11	8.2 × 10^−4^	6	4.5 × 10^−4^
**X**		7030	5	2.1 × 10^−3^	2	8.5 × 10^−4^
**C**		9718	5	1.5 × 10^−3^	2	6.1 × 10^−4^

Although there were fewer available sequences for analysis in treated patients we found 21 premature stops in the C, P and X genes for this group, a significantly elevated frequency compared with untreated patients (S1 Table; Fisher exact test: *p* < 0.001). The estimated mutation rate for treated patients was 1.1 × 10^−3^ m/n/c, i.e. 12-fold higher than in untreated patients. In the treated group, we also found that several stop codons appeared repeatedly in the same or different patients, suggesting non-lethality or the presence of mutational hotspots. Removing these cases, there were still 10 stop codons in treated patients, yielding a mutation rate estimate of 5.2 × 10^−4^ m/n/c, a value still 6-fold higher than in untreated patients (Fisher exact test: *p* < 0.001).

**Fig 2:**
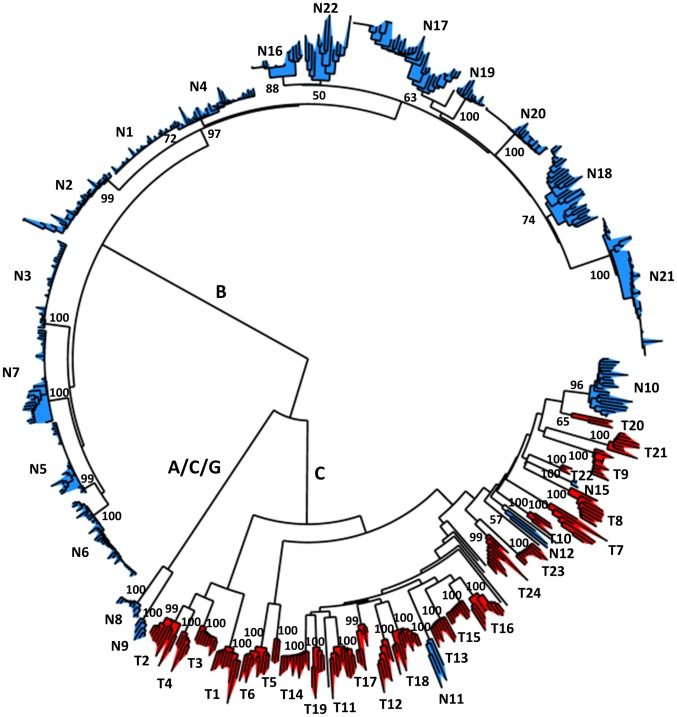
Phylogenetic analysis of HBV sequences used in this study. A neighbor-joining containing the 621 full-length sequences used is shown. Patient names are as in Table 1. Sequences from untreated patients are marked in blue and those from treated patients in red. For each indicated patient, numbers show the bootstrap value of the node that delimitates the sequences from this patient. Sequences from patients N16 to N22 correspond to a study in which several family members were studied and are partially intermingled (not shown). Patients N13 and N14 are not shown because their sequences did not form well-defined monophyletic groups. Letters in the center of the tree indicate the viral genotype.

These results suggest that lamivudine/adefovir treatment has a marked effect on the mutation rate of HBV in vivo. However, several possible confounders may have affected our conclusion. First, we found limited information about patient infection times, but this should not represent a major source of error because the lethal mutation rate estimation method is not sensitive to the number of viral generations elapsed. Second, some of the observed mutations might have been introduced during PCR amplification. However, for all patients except three untreated, high-fidelity polymerases with similar error rates were used (Table 1), making it unlikely that the observed differences were driven by PCR errors. Third, as mentioned above, stop-codon counts were not homogeneous among genes. However, the fact that we used always full-length genome sequences should make differences between treated and non-treated patients robust to among-gene variations. Fourth, all treated patients were infected with genotype C, whereas most sequences from untreated patients belonged to genotype B (Fig 2). Therefore, we cannot rule out the possibility that the observed differences in mutation rate were driven by a higher spontaneous mutation rate of HBV genotype C compared to genotype B. To address this issue, we downloaded 1481 molecular clone sequences of a region of the preC/C reading frames from a single study in which untreated patients infected with HBV genotype B or C were enrolled [47]. After genotyping the sequences and removing the preC region, we found four unique premature stops in the 724 sequences belonging to genotype B, versus one such stop in the 757 sequences belonging to genotype C, hence arguing against a higher spontaneous mutation rate in genotype C. Therefore, the conclusion that lamivudine/adefovir treatment increases the mutation rate of HBV appears to be robust to several sources of error and bias.

**Fig 3:**
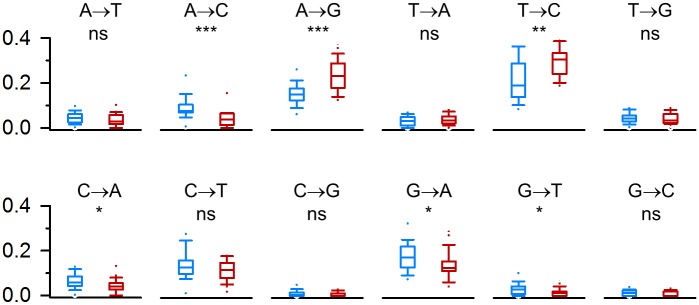
HBV mutational spectrum in untreated patients and in patients undergoing lamivudine/adefovir treatment. The mutational spectrum was obtained in sequences derived from untreated (blue) or treated (red) patients by scoring all intra-patient SNPs along the HBV genome. Box plots indicate the relative contribution of each substitution type to the total SNPs found. Lower and upper box limits indicate percentiles 25^th^ and 75^th^, respectively, and the middle line shows the median. Whiskers show the 10^th^ and 90^th^ percentiles, and outlying points are plotted individually. Differences between treated and untreated patients in the frequency of each substitution type were assessed by a Mann-Whitney non-parametric test (***: p < 0.001; **: 0.001 < p < 0.01; *: 0.01 < p < 0.05; ns: non-significant).

To test whether treatment modified the type of mutations produced by HBV, we scored all intra-patient single-nucleotide polymorphisms (SNPs) in the 621 full-length molecular clone sequences used above for mutation rate estimation by comparing each sequence with the consensus sequence of the patient. In total, we found 3342 intra-patient SNPs along the HBV genome, and we calculated the percentage contribution of each type of nucleotide substitution to the total number of SNPs found in each patient (mutational spectrum). The most frequent type of SNP was T→C, followed by the other base transitions A→G, C→T, and G→A, whereas base transversions were less frequent. However, the mutational spectrum differed between untreated and treated patients (Fig 3). Lamivudine/adefovir treatment was associated to a greater relative abundance of A→G SNPs (Mann-Whitney test: *p* < 0.001) and the complementary T→C changes (*p* = 0.001). All other types of nucleotide substitutions were not significantly modified in treated patients, or showed lower relative abundance.

## Discussion

Our spontaneous mutation rate estimate of 8.7 × 10^−5^ m/n/c should be taken as an upper-limit for several reasons. First, a fraction of the observed premature stop codons may be artefacts introduced during PCR amplification, cloning, or sequencing. Second, hypothetical read-through of some stop codons may prevent their lethality. Third, the lethal effects of some stop codons may be rescued by trans-complementation in cells co-infected with other, functional variants of the virus, leading to higher-than-expected frequencies of premature stop codons. Trans-complementation may explain the large differences among genes in the frequency of stop codons, which were >20-fold higher in the S gene than in the polymerase gene P in untreated patients (Table 2). Since reading frames are strongly overlapped in HBV, we do not interpret these variations as real changes in mutation rate along the HBV genome. We suggest that the lethality of some stop codons is more likely to be rescued by trans-complementation in the surface envelope than in the RT because HBV-infected cells produce a large excess of surface proteins, which are released to the serum in the form of empty, non-infectious sub-viral particles [48,49]. In contrast, the viral polymerase is produced in smaller amounts and should be more tightly associated with the viral genome, making trans-complementation less likely. Considering this, the mutation rate obtained for the P gene of 2.7 × 10^−5^ m/n/c might be more accurate than the value obtained for other genes. Interestingly, among-gene stop-codon frequency variation was smaller in treated patients than in untreated patients (Table 2), potentially indicating a lower effect of trans-complementation. The co-infection probability increases with viral load and, hence, should tend to be higher in untreated patients than in treated patients.

Although it is commonly accepted that the main source of spontaneous mutations in HBV is the low replication fidelity of the viral polymerase, the HBV genome is subject in vivo to genome editing by cellular enzymes of the apolipoprotein B mRNA editing enzyme, catalytic polypeptide-like 3 (A3) family, which produce in G→A substitutions in the viral genome [50,51]. Of the five premature stop codons found in X, C, and P sequences derived from untreated patients, one involved a G→A substitution (TGG→TGA), two involved C→T substitutions (unlikely to be driven by A3), and two involved base transversions (clearly not driven by A3). This suggests that A3 is not the main source of spontaneous mutations in HBV. In recent work with HIV-1, we have shown that A3 activity contributes approximately half of the spontaneous mutations found in plasma, but 98% of those found in peripheral blood mononuclear cells [33]. Here, the vast majority of sequences analyzed were obtained from plasma samples. Analysis of sequences from liver tissue may contribute to better elucidate whether A3 activity contributes significantly to the total rate of spontaneous mutation of HBV.

Lamivudine/adefovir treatment increased significantly the frequency of premature stop codons. This effect was strongest in the P gene (>15-fold) and mildest in the S gene (approximately twofold). We argue that the actual mutagenic effect of lamivudine/adefovir is best reflected by the P gene because estimation bias is probably less pronounced than in the other genes, particularly S. Our analysis of the full set of SNPs revealed a specific enrichment in A/U→G/C substitutions in HBV sequences derived from treated patients. Recent work in mice chronically exposed to lamivudine has shown the same mutagenic pattern, as determined by sequence analysis of the highly variable D-loop region of mitochondrial DNA isolated from cortical neurons [52]. The fact that the mutational spectrum observed in treated patients was similar to that found in mouse mitochondrial DNA suggests a direct mutagenic effect of lamivudine/adefovir, as opposed to an alternative scenario in which HBV polymerase variants with modified mutational properties would have evolved in response to treatment. Since lamivudine is a cytosine analogue, it is expected to be incorporated into DNA by base-pairing with guanosine, but it is unclear how this should lead to A/U→G/C substitutions. Adefovir is an adenosine analogue and may cause A/U→G/C substitutions after being incorporated into DNA by base-pairing with thymidine, followed by incorporation of cytosine by base-pairing with adefovir, or through other pathways. Further work will be required to elucidate the mechanistic basis of the observed mutagenic effects.

Our results clearly show that lamivudine/adefovir treatment increases the HBV mutation rate above 10^−4^ m/n/c in each viral gene (Table 2). For a per-site mutation rate of 5.2 × 10^−4^ m/n/c, approximately 1.5 new mutations should be produced per genome after a single cell infection cycle. This suggests that, in treated patients, HBV produces mutations faster than many RNA viruses. For comparison, the spontaneous mutation rate of hepatitis C virus (HCV) *in vivo* estimated by the lethal mutation method is on the order of 0.3–1.2 × 10^−4^ m/n/c, and ribavirin/interferon treatment increases this rate between two- and three-fold [21,32]. It is noteworthy that the effect of ribavirin/interferon treatment on the HCV mutation rate appears to be less marked than that of lamivudine/adefovir treatment for HBV. Previous work has suggested that modest increases in the mutation rate of RNA viruses can situate them close to the maximum tolerable mutation rate, or error threshold [53]. In the presence of treatments, the estimated mutation rates of HBV and HCV are similar and, therefore, we speculate that treatment may also situate HBV close to the error threshold, particularly if we consider the high degree of gene overlap shown by this virus, in which many single-point mutations modify the sequence of two proteins simultaneously. However, this extremely high mutation rate may also boost viral evolvability and promote the rapid emergence of drug-resistance and immune escape mutants. Previous work has demonstrated that, in HBV and RNA viruses, high fidelity variants can evolve in response to nucleoside analogue mutagenesis [13,54–58]. If a similar process occurs in vivo for HBV, it seems not to be sufficient to compensate for the mutagenic effect of lamivudine/adefovir treatment. Future work may further elucidate how HBV evolves in response to treatment-induced mutagenesis. Ideally, this should be addressed in longitudinal studies that include samples before and after the onset of treatment, or using HBV genotype-matched patients in the case of cross-section studies. Similarly, sequencing at both the acute and chronic diseases stages for patients with known infection times would allow testing whether the HBV mutation rate evolves throughout the course of infection.

## Supporting Information

S1 Table List of premature stop codons indicating the patient, gene, genome position, type of stop and accession number, in Excel format. (XLSX)
